# Temperature- and thickness-dependent elastic moduli of polymer thin films

**DOI:** 10.1186/1556-276X-6-243

**Published:** 2011-03-22

**Authors:** Zhimin Ao, Sean Li

**Affiliations:** 1 School of Materials Science and Engineering, The University of New South Wales, Sydney, NSW 2052, Australia

## Abstract

The mechanical properties of polymer ultrathin films are usually different from those of their counterparts in bulk. Understanding the effect of thickness on the mechanical properties of these films is crucial for their applications. However, it is a great challenge to measure their elastic modulus experimentally with *in situ *heating. In this study, a thermodynamic model for temperature- (*T*) and thickness (*h*)-dependent elastic moduli of polymer thin films *E*_f_(*T*,*h*) is developed with verification by the reported experimental data on polystyrene (PS) thin films. For the PS thin films on a passivated substrate, *E*_f_(*T*,*h*) decreases with the decreasing film thickness, when *h *is less than 60 nm at ambient temperature. However, the onset thickness (h*), at which thickness *E*_f_(*T*,*h*) deviates from the bulk value, can be modulated by T. h* becomes larger at higher *T *because of the depression of the quenching depth, which determines the thickness of the surface layer δ.

## Introduction

As devices are being developed with a view towards making them smaller, thinner and lighter in dimension, thin polymer films are found to be in more stringent demand in various applications, such as diffusion barriers, dielectric coatings, electronic packing, and so on [1]. Therefore, understanding the elastic modulus in confined geometries, such as in thin films, is critical to the stability of the structures of the actual devices. A growing demand exists for the determination of the mechanical properties of thin films and coatings at a rapid pace. Recent researches primarily focusing on the confinement effect of the glass-transition temperature *T*_g _in thin films [2-7], have presented inconsistent results. It is believed that such a phenomenon might be attributed to the surface and interfacial effects. However, despite its technological importance, the corresponding elastic modulus of the confined thin polymer films has yet to be fully characterized to the same extent as *T*_g _effects have been done.

Direct experimental measures of the ultrathin material modulus have proven to be difficult since the presence of stiff substrate tends to interfere with the measurements [8]. With regard to the impact of confinement on the elastic modulus of soft materials, there is no agreement. This occurrence was caused by the various measurement strategies [9], similar to the initial polymer thin film *T*_g _measurements [4,6]. The different trends of the elastic modulus are due to the different interfacial interactions between probe and the polymer surface. Thus, noncontact mechanics measurement, which does not disturb the free surface, appears to be potentially advantageous for determining the modulus of polymer films. Recently, a wrinkle-based metrology was developed to measure the elastic properties of thin polymer films [10]. In surface wrinkling measurements, to determine the modulus of thin polymer films, a wrinkling instability is utilized to induce compression of a stiff film bonded to a compliant substrate. The film modulus *E*_f _is determined based on the formula relating the substrate modulus *E*_sub_, the film thickness *h*, and the wrinkling wavelength λ: . At room temperature, the deduced elastic modulus of the films decreases with decreasing film thickness for ultrathin polymer films (thickness less than 30 nm) [11]. In order to understand better the physical nature of the thickness dependence of the deduced elastic modulus in ultrathin films, a bilayer model was proposed to account for the surface effect on the wrinkling associated with the surface of a soft layer [12].

In the most recent research, the elastic moduli of a series of poly(methacrylate) films with widely varying bulk glass-transition temperature (*T*_gb_) as function of thickness at ambient temperature were measured by wrinkle-based metrology. A decrease of the modulus was found in all ultrathin polymers films (< 30 nm) with the onset of confinement effects shifting to larger film thicknesses as the quench depth into glass state (*T*_gb _- *T*) decreases, where *T *is the measured temperature [8]. In other words, the quench depth affects the extent of the size dependence. To have a better clarification of the quench-depth effect on the elastic modulus of thin polymer films and the nature of the glass transition of polymers, *T *was considered as a variant to obtain the temperature-dependent elastic modulus of thin films. However, the *in situ *heating of a polymer-substrate system covering all the processing and characterization steps is impractical [8]. Therefore, in this study, a model will be developed to investigate the temperature- and thickness-dependent elastic moduli of thin polymer films based on both the bilayer and the size-dependent glass-transition temperature thermodynamic models. The results of this new proposed model are then verified with experimental data that were obtained by wrinkle-based metrology for thin polystyrene (PS) films with different molar weights (*M*_w_).

## Theoretical model

It is well known that the atomic or the molecular structure on a surface is different from the bulk structure in solids. As a result, many materials' properties (e.g., mass density, electrical conductivity, elastic modulus, etc.) on the surface differ from their bulk counterparts. Such a difference is negligible for large-scaled structures. For nanostructures, however, the surface-to-volume ratio is large, and the surface effects can be significant. For thin polymer films of interest in this study, one may assume that a surface layer exists with different elastic moduli. The thickness of the surface layer δ may vary from say one atomic layer for crystalline materials [13-15] to a few nanometers for polymers [16,17]. In the surface layer, lesser density, larger mobility, and softening were found for polymer films [5,17,18]. Therefore, a bilayer model was proposed to describe the elastic modulus of thin polymer films *E*_f _[12]. In brief, the model consists of a polymer film with thickness *h*, containing two distinct moduli. The surface layer of the film has a modulus *E*_sur _and a finite depth of δ underneath the atmosphere/solid interface. This depth was considered to be independent of the film thickness. The remainder of the film (*h*-δ) exhibits a bulk-like modulus (*E*_bulk_). The bilayer model can be written as [12](1)

*E*_sur _was considered a few orders of magnitude smaller than the corresponding *E*_bulk _[11,12]. On the other hand, it is known that temperature influences the elastic properties of solids [13,19]. In general, the elastic moduli of solids would decrease at high temperature due to the weakening of the interactions between atoms or molecules induced by the thermo-expansion of solids [14]. However, Equation 1 ignores the temperature effect, although the mechanical properties of amorphous polymers are slightly influenced by temperature in the glass region. The mechanical properties would change significantly as the temperature approaches the *T*_g _[20]. In addition, the bilayer model considered that δ is independent of *h*. However, computer simulations have found that a thinner polymer film has a thicker surface soft layer at a given temperature, and the soft layer would extend to almost the whole film at *T*_g _[20]. Recent research has shown that the following relationship exists for bulk polymers: [8]. Thus, δ is related to the quench depth (*T*_gb _- *T*). In the nanometer-scale range, the relationship is considered to be still valid since polymer films and corresponding bulk polymer have similar thermodynamic behaviors [18,20]. In addition, simulations have noted that near *T*_g _the thickness of the free surface layer can nearly extend throughout the thin film [20]. Therefore,(2)

The glass-transition temperature of thin polymer films *T*_g_(*h*) is also dependent on the thickness [2-7]. Therefore, after considering temperature effects for *T *<*T*_g_(*h*), the bilayer model can be modified as(3)

It is noted that the temperature effect on the film thickness is ignored in Equation 3 as the thermal expansion is relatively small compared with the thickness variation here. In this case, *T*_g_(*h*) can be determined by [4,5](4)

where *h*_0 _= 2*c*ξ, with ξ being the correlation length for the intermolecular cooperative rearrangement; *c *is a parameter related with the surface and interface: *c *= 1 for free-standing thin films or supported thin film with strong interaction between the polymer and the substrate, such as hydrogen bonding; and *c *= 1/2 for a supported thin film with weak interaction between the polymer and the substrate, such as van der Waals force, which is equivalent to the disappearance of the interface. α_s _= [2Δ*C*_pb_/3*R*]+1, where *R *is the ideal gas constant, and Δ*C*_pb _is the heat-capacity difference between the bulk glass and the bulk liquid at *T*_gb_. α_i _= α_s_*E*_s_/*E*_i _with *E*_s _and *E*_i _being the bond strength at the surface and interface, respectively.

## Results and discussion

The theoretical model was applied to the PS thin films to verify the newly developed temperature- and thickness-dependent elastic modulus model. First, *E*_bulk_(*T*) and *T*_g_(*h*) should be determined for the PS films. It is known that the elastic properties decrease almost linearly in the glass state. They present a very strong temperature-dependent behavior near *T*_g _[8,19]. In addition, generally the glass transition occurs from *T*_g_-50 K for bulk polymers. Therefore, *E*_bulk_(*T*) for bulk PS at *T *<*T*_g_-50 K is a linear function, which can be deduced from the experimental data obtained from *E*_bulk_(*T*) = -0.00189*T *+ 4.558 [19]. The result is also in agreement with another experimental result where the elastic modulus of bulk PS, *E*_bulk _= 4.0 GPa at *T *= 294 ± 3 K [12]. *E*_sur _was considered much smaller than the corresponding *E*_bulk_; it is about 0.1 GPa for PS [12].

**Figure 1:**
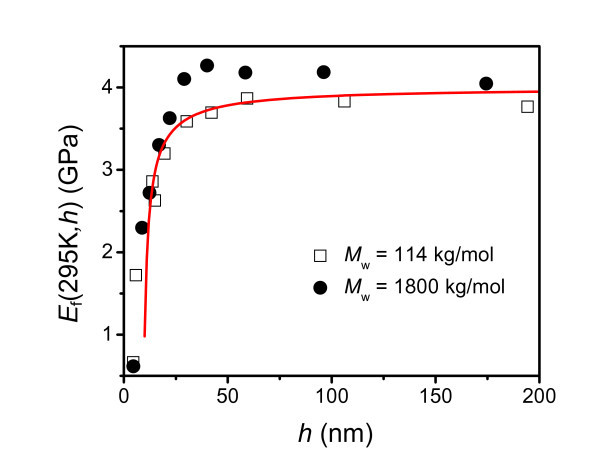
**The thickness-dependent elastic modulus of PS thin films at *T *= 295 K**. The symbols circle and square are the experiment results for molar weight *M*_w _= 1800 and 114 kg/mol, respectively [12]. The solid curve is plotted with the calculation results obtained from Equation 3, where the parameters that determine the *T*_g_(*h*) in Equation 4 are *c *= 1/2, *h*_0 _= 5 nm, Δ*C*_pb _= 30.7 J mol^-1 ^K^-1 ^= 1.919 J g atom^-1 ^K^-1^, and *T*_gb _= 375 K [4].

Figure 1 shows the thickness dependence of the elastic modulus of PS thin films at *T *= 295 K that was obtained from Equation 3 using expression (4). The parameters needed for the PS thin films are given in the caption of Figure 1. In this figure, our results are compared with the corresponding experimental results for two different molar weights. These results are well in agreement with each other. The modulus for thick films (> 100 nm) was found to be independent of the film thickness, whereas the elastic modulus decreases with the film thickness when the thickness is less than 60 nm. A similar thickness-dependent behavior was also found for several other polymer films, such as poly(methyl methacrylate) (PMMA), poly(ethyl methacrylate) (PEMA), and poly(isobutyl methacrylate) PiBMA [8,11]. The depression of the elastic modulus for thin films is a consequence of the soft surface layer, whose relative importance increases as the surface-to-volume ratio increases.

**Figure 2:**
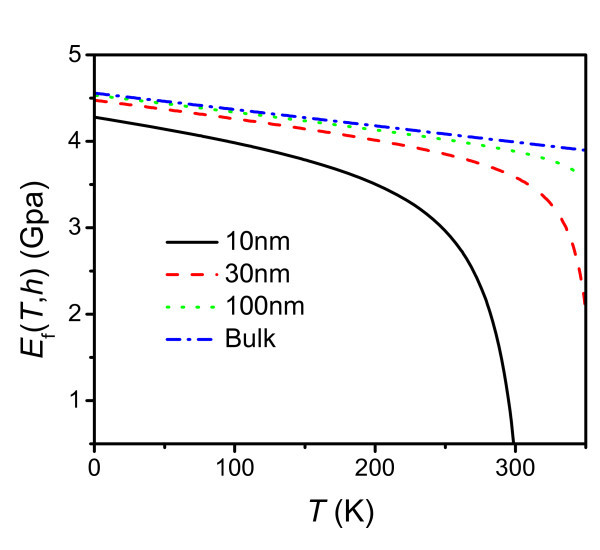
**The temperature-dependent elastic modulus of PS thin films with *h *= 10, 30, and 100 nm and bulk obtained from Equation 3**.

Using Equation 3, the temperature dependence of elastic modulus of PS thin films with *h *= 10, 30, and 100 nm and bulk are shown in Figure 2. It is noted that the elastic modulus decreases as *T *increases for all the films. At low temperatures, there is a nearly linear relationship between the elastic modulus and temperature. However, the elastic modulus decreases steeply at the onset of approximately 210, 300, and 350 K for 10, 30, and 100 nm PS thin films, respectively, while the glass-transition temperatures are 275, 347, and 368 K for 10, 30, and 100 nm, respectively, based on Equation 4. The differences between the onset point and *T*_g _are 65, 47, and 18 K, respectively, for the films of three different thicknesses, which imply that the modulus decreases greatly in the glass-transition temperature region, and this region is more extended as the thickness decreases, consistent with the available experimental data in published literatures [19,21,22].

**Figure 3:**
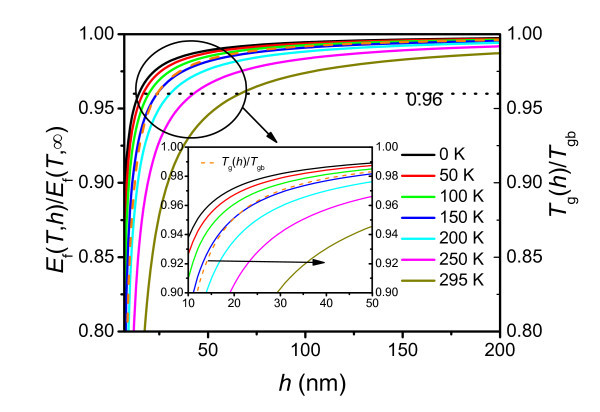
**The thickness-dependent elastic modulus of PS thin films at different temperatures**. The inset enlarges the encircled region to show the good match of *T*_g_(*h*)/*T*_gb _and *E*_f_(*T*,*h*)/*E*_f_(*T*,∞).

**Figure 4:**
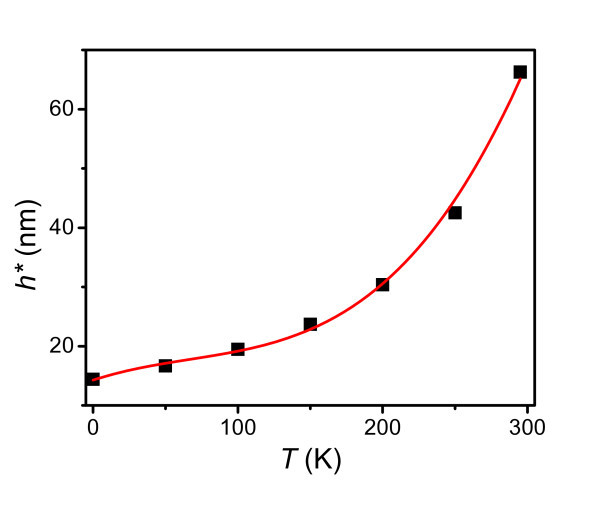
The onset thickness h* of PS films on the passivated substrate at different temperatures.

Most recently, the deviation of the elastic modulus from its bulk value was studied for thin glassy polymer films with different glass-transition temperatures at ambient temperature. These results suggested that the deviations are significantly influenced by the quench depth into the glass (*T*_gb _- *T*) [8]. To induce the different quench depths, the temperature *T *is varied in this study. Figure 3 plots the thickness-dependent elastic moduli of PS thin films at different temperatures. For clearly demonstrating the size effect, a relative value of *E*(*T*,*h*)/*E*(*T*,∞) is taken as the ordinate in the figure. From Figure 3, it is found that the size effect is more significant at high temperatures. Recent research has reported that the elastic modulus and glass-transition temperature deviate from the corresponding bulk values at the same thicknesses for poly(*n*-propyl methacrylate) (PnPMA) thin film on poly(dimethylsiloxane) (PDMS) substrate [8]. In Figure 3, *T*_g_(*h*)/*T*_gb _for PS thin films on a passivated substrate is shown by the dashed curve, which is obtained by Equation 4. We find that the functions *T*_g_(*h*)/*T*_gb _and *E*_f_(150,*h*)/*E*(150,∞) behave in a similar manner as a function of *h*. Also, the thicknesses at which the two functions deviate from the corresponding bulk values are almost the same, as in the case of the PnPMA/PDMS system. Considering a criterion that the deviation of the modulus starts when *E*(*T*,*h*)/*E*(*T*,∞) ≈ 0.96, the critical film thickness h*, at which the deviation starts for different temperature, is shown in Figure 4. Note that h* increases as *T *increases. In other words, at high temperatures, the size effect is more important, and it can be tuned by the application temperature. Therefore, in actual applications, to avoid decreasing the strength of thin films, one should make sure that the film thickness is larger than h* for a given temperature. The temperature dependence of h* is the consequence of the depression of quench depth when the temperature is near the glass-transition region.

**Figure 5:**
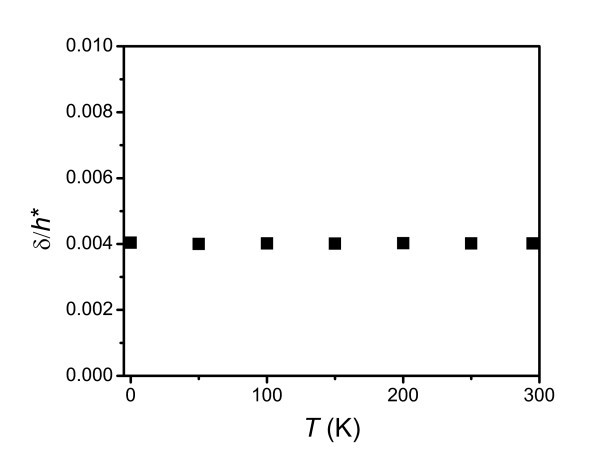
The ratio δ/h* as a function of temperature.

**Figure 6:**
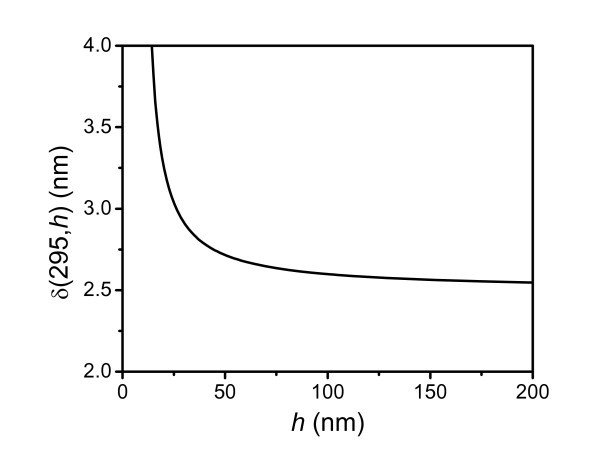
**Thickness dependence of the surface layer δ(295 K,*h*) of PS thin films**.

The effect of the application temperature on the elastic modulus of thin polymer films is seen from the plots of Figure 5, showing the ratio δ/h* as a function of temperature. It is noted that this ratio is almost a constant, i.e., approximately 0.004. It is known that the size effect is determined by δ/*h*, where δ is usually considered as independent of *h *at a given temperature [8,12]. However, Figure 6 shows the surface thickness of PS films of different thicknesses at 295 K as obtained from Equation 2. Thus, δ depends on *h *and increases as *h *decreases. According to Equation 2, it is known that δ is related to *T*_g_(*h*), which is dependent on size and can be determined by Equation 4. In the case of PS films on the passivated substrate, such as on PDMS, where there is no strong interaction between the polymer films and the substrate, *T*_g_(*h*) decreases with decreasing *h*. Therefore, δ(*h*) increases as *h *decreases due to the depression of *T*_g_(*h*) at a given temperature. On the other hand, the mobility of the polymer film surface layer can be experimentally investigated by nanoparticle embedding [17,23] and fluorescence methods [7]. Both methods reported that the surface layer has a thickness of several nanometers, which is consistent with the result of Figure 6.

## Conclusion

A theoretical model for the temperature- and thickness-dependent elastic modulus *E*_f_(*T*,*h*) was established for amorphous polymer thin films to investigate the dominance of this mechanical property of PS films at nanometer scale. We found that at ambient temperature, *E*_f_(*T*,*h*) of PS thin films on the passivated substrate decreases as *h *decreases when *h *is thinner than 60 nm, while *E*_f_(*T*,*h*) is nearly independent on *h *for *h *> 60 nm. Furthermore, a significant thickness effect can be induced by the temperature. The onset of thickness, at which *E*_f_(*T*,*h*) deviates from the bulk value, is dependent on temperature and is larger at high temperature. At a certain temperature, *E*_f_(*T*,*h*) exhibits the same size-dependent trend as *T*_g_(*h*), which is associated with the quench depth of *T*_g_(*h*) - *T*. Except for the temperature effect, the thickness of the surface layer also depends on *h*, and it increases as *h *decreases due to the size-dependent glass-transition temperature *T*_g_(*h*). Therefore, *E*_f_(*T*,*h*) of the thin films can be determined using the developed model, thus providing references for the applications of polymer thin films.

## Abbreviations

PDMS: poly(dimethylsiloxane); PEMA: poly(ethyl methacrylate); PiBMA: poly(isobutyl methacrylate); PS: polystyrene; PMMA: poly(methyl methacrylate); and PnPMA: poly(*n*-propyl methacrylate).

## Competing interests

The authors declare that they have no competing interests.

## Authors' contributions

ZA developed the model and drafted the manuscript. SL co-drafted the manuscript. All authors read and approved the final manuscript.
